# The g.4290 C>G Polymorphism in the *FADS2* Gene Modifies the Fatty Acid Profile of the *Pectoralis Superficialis* Muscle of Ross 308 Broiler Chickens

**DOI:** 10.3390/ani12151882

**Published:** 2022-07-22

**Authors:** Urszula Kaczor, Sebastian Sawicki, Joanna Nowak, Julia Gabryś, Jakub Jurczyk, Dorota Wojtysiak, Katarzyna Połtowicz

**Affiliations:** 1 Department of Nutrition, Animal Biotechnology and Fisheries, Faculty of Animal Science, University of Agriculture in Kraków, 21 Mickiewicza Ave., 31-120 Cracow, Poland; sebastian.sawicki@student.urk.edu.pl (S.S.); julia.gabrys@student.urk.edu.pl (J.G.); jakub.jurczyk@doctoral.uj.edu.pl (J.J.); 2 Department of Poultry Breeding, National Research Institute of Animal Production, 32-083 Balice, Poland; joanna.nowak@iz.edu.pl; 3 Department of Genetics, Animal Breeding and Ethology, Faculty of Animal Science, University of Agriculture in Kraków, 21 Mickiewicza Ave., 31-120 Cracow, Poland

**Keywords:** *FADS2*, *FABP4*, gene polymorphism, meat quality, fatty acids, broiler chicken

## Abstract

**Simple Summary:**

The fatty acid profile of meat of various species of farm animal determines the health-promoting properties of meat products. Primates, like other mammals, are not able to synthesize essential fatty acids, but can convert essential fatty acids provided with food to long-chain fatty acids with 20 and 22 carbon atoms. Therefore, essential fatty acids supplied in the diet play a huge role in human nutrition, including meat products. The effect of the g.4290 C>G substitution in the *FADS2* gene and g.285 C>T in the *FABP4* gene on carcass quality, meat quality, and fatty acid profile of the *pectoralis superficialis* muscle of broiler chickens was analyzed. A significant influence of g.4290 C>G in the *FADS2* gene on the *pectoralis superficialis* muscle fatty acid profile was demonstrated. The studied polymorphism determined the saturated fatty acids and monounsaturated fatty acids, as well as the content of docosahexaenoic and eicosapentaenoic acids important in human nutrition. Polymorphisms in the *FADS2* gene *locus* affect animal fat characteristics and can be used as genetic markers for poultry meat.

**Abstract:**

The effect of the g.4290 C>G substitution in the *FADS2* gene and g.285 C>T in the *FABP4* gene on carcass quality, meat quality, and fatty acid profile of the *pectoralis superficialis* muscle of 238 male broiler chickens reared up to 45 days of age was analyzed. A significant influence of g.4290 C>G in the *FADS2* gene on the *pectoralis superficialis* muscle fatty acid profile was demonstrated. Chickens with the GG genotype were characterized by the highest content of conjugated linoleic acid, amino acids, eicosapentaenoic acids, docosapentaenoic acid, docosahexaenoic acids. and the lowest value of the linoleic acid/alpha-linolenic acid ratio. The *FABP4* polymorphism determined only the content of C18:1n-9, C18:2n-6 and docosahexaenoic acid. There was no effect of the studied genotypes on final body weight, carcass quality traits, or quality of broiler pectoral muscles.

## 1. Introduction

The fatty acid profile of meat of various species of farm animals determines the health-promoting properties of meat products. There are significant interspecies differences in the content of individual fatty acids, which is determined, among other things, by nutrition, animal breed, growth rate, location of meat tissue, and variation within genes involved in fatty acid metabolism [1,2,3,4,5].

Mammals are dependent upon either consumption of preformed >20 carbon PUFA in their diet or conversion of linoleic acid (LA, 18:2n-6) and alpha-linolenic acid (ALA; C18:3n-3) to longer-chain, more unsaturated fatty acids to meet their PUFA requirements. The n-3 and n-6 essential fatty acids accumulate in tissue phospholipids in the form of 20- and 22-carbon highly unsaturated fatty acids (HUFA) that mediate and monitor the impact of food choices on health. In a human, eating food with nutrients that maintain less than 50% n-6 in HUFA is an effective form of primary prevention of cardiovascular disease. Therefore, essential fatty acids supplied in the diet play a huge role in human nutrition, including meat products [6].

Fatty acid desaturases form the Fads1–Fads8 protein family, whose function is to introduce a double bond into the hydrocarbon chain of fatty acids and other lipids. Delta-5(D5D) and delta-6 desaturases (D6D), encoded by the *FADS1* and *FADS2* genes, respectively, are key enzymes whose activity is required for the synthesis of LC-PUFA, esterified in membrane phospholipids, that influence biological membrane permeability [7]. Delta-5 desaturase is necessary for the conversion of dihomo-γ-linolenic acid (DGLA, C20:3n-6) to arachidonic acid (ARA, C20:4n-6) and C20:4n-3 to eicosapentaenoic acid (EPA, C20:5n-3). The transmembrane delta-6 desaturase enzyme has the ability to convert LA and ALA into γ-linolenic acid (GLA, C18:3n-6) and stearidonic acid (STA, C18:4n-3). The presence of a number of desaturases and elongases means that GLA and STA can be converted to long-chain polyunsaturated fatty acids, i.e., ARA, which is a precursor of eicosanoids: EPA (C20:5) and docosahexaenoic acid (DHA, C22:6). Delta-6 desaturase is a factor that limits the activity of ALA in EPA synthesis [8,9].

Fatty acid binding proteins (FABP) belong to intracellular lipid-binding proteins (iLBPs) found in vertebrates and invertebrates. Nine tissue-cytoplasmic fatty acid binding proteins were identified based on the tissue from which they were isolated. Given the similarity of amino acid sequences, the FABP family can be divided into three groups: H-FABP (FABP3), B-FABP, and M-FABP. The second group contains typical for adipocytes—A-FABP (*FABP4*) and epidermal—E-FABP, while the third group includes lipid-binding proteins in the liver—L-FABP and intestine—I-FABP [10]. The *FABP4* protein, present predominantly in adipocytes and macrophages, is responsible for the uptake and storage of fatty acids in the adipose tissue [11]. *FABP4* plays an important role in the development of insulin resistance and atherosclerosis, and its increase in blood levels may be associated in humans with obesity, diabetes, hypertension, and cardiovascular diseases [12]. *FABP4*-deficient mice show higher body weight with decreased insulin resistance [13].

In hen, the *FADS2* gene contains 12 exons and is located on chromosome 5. The *FABP4* gene has 4 exons separated by 3 introns and is located on chromosome 2.

Polymorphisms in the *FADS2* gene *locus* affect animal fat characteristics and can be used as genetic markers of bovine [14,15] and poultry meat [4]. Polymorphisms in the human *FADS2* gene determine the profile of omega n-3 and omega n-6 fatty acids in white adipose tissue. Risk-allele carriers show decreased D6D activity and consequently lower levels of LC-PUFA. Polymorphisms that may be associated with quality traits of beef or pork have been localized in the *FABP4* gene [16,17,18].

The aim of the study was to determine the effect of single-nucleotide polymorphisms (SNPs) in the regulatory region of the *FADS2* gene and in exon 1 of the *FABP4* gene on carcass quality, meat quality, and fatty acid profile of the *pectoralis superficialis* muscle in the broiler chicken line Ross 308.

## 2. Materials and Methods

### 2.1. Ethics Approval

All conducted chicken trials were approved by the Approving Experiment Committee of National Research Institute of Animal Production (Balice, Poland) and the national authority according to the Polish Act on the Protection of Animals Used for Scientific or Educational Purposes of 15 January 2015 (which implements Directive 2010/63/EU of the European Parliament and the Council of 22 September 2010 on the protection of animals used for scientific purposes). Moreover, all procedures were following the guidelines and regulations of the local Krakow Ethics Committee for Experiments with Animals.

### 2.2. Materials

The 1-day-old sexed Ross 308 chicks (cockerels) (*n* = 238) were placed in pens with an area of 3.5 m^2^ each and reared to 45 days of age on litter under optimal environmental conditions, with a stocking density not exceeding 33 kg/m^2^. All birds were fed *ad libitum* with complete starter (1–21 days), grower (22–35 days), and finisher (36–45 days of age) feed mixes containing 22, 20.5, and 20.5% crude protein and 2990, 3130, and 3130 kcal/kg metabolizable energy, respectively, and had unlimited access to drinking water. At 45 days of age, all birds were sacrificed by decapitation after 10 h of fasting.

The chickens were reared at the experimental poultry farm of the National Research Institute of Animal Production located in Aleksandrowice, Poland.

### 2.3. Methods

#### 2.3.1. Blood Sample Collection

The research material (venous blood) was collected from the broilers during the bird slaughter on the 45th day of their life, into 5 mL tubes (MedLab Products, Raszyn, Poland) with K3EDTA anticoagulant. Until analysis, the samples were stored in a freezer at −28 °C.

#### 2.3.2. Evaluation of Dressing Percentage and Carcass Quality

After 24 h of cooling, carcass dressing percentage was estimated as the ratio of chilled carcass with neck and abdominal fat to live body weight. All carcasses were subjected to simplified slaughter analysis. The proportion of breast muscle, leg muscle (thigh and drumstick), abdominal fat, and edible giblets was calculated as a percentage of the cold carcass weight.

#### 2.3.3. Evaluation of the Quality Parameters of the Pectoralis Superficialis Muscle

*Pectoralis superficialis* muscles were dissected from the chilled carcasses and evaluated for technological properties (pH, color, thermal loss, shear force), and the fatty acid profile of intramuscular fat.

All technological meat characteristics were determined following the method described by Połtowicz et al. [19].

#### 2.3.4. Fatty Acid Profile Determination

Intramuscular fat of the *pectoralis superficialis* muscle was extracted using the method of Folch et al. [20]: a mixture of chloroform (pure for analysis (p.a.) and methanol p.a. (*v/v*, 2/1). Half a milliliter of 0.5 N KOH in methanol was added to the fat (10 mg) and heated to 85 °C, then 1 mL of 12% BF3 in methanol was added and reheated to 85 °C. After cooling to room temperature, 1 mL of hexane and 5 mL of saturated NaCl solution were added. One microliter of the mixture was injected onto the chromatograph. Individual fatty acid methyl esters were identified by comparison to the standard mixture of (Supelco 37 component FAME Mix, Sigma-Aldrich Co., St. Louis, MO, USA) A TRACE GC ULTRA chromatograph (Thermo Electron Corporation, Milano, Italy) was used with the following parameters: flame ionization detector (FID) with a temperature of 250 °C; dispenser −220 °C; furnace −160 °C for 3 min, heating to 210 °C for 3 min, and next held 210 °C for 35 min. A SUPELCOWAX 10 column with dimensions of 30 m × 0.25 mm × 0.25 µm. Carrier gas—helium 1 mL/min, split flow 10 mL/min. Each sample was analyzed in two repetitions. Results of FA content are presented as the percentage of total fatty acids (%TA) detected and calculated with ChromQuest 4.1 software (Thermo Electron, Milan, Italy). The individual fatty acid peaks were identified by comparison of retention times with those of known mixtures of standard fatty acids (FAME, Sigma-Aldrich CO., St. Louis, MO, USA) run under the same operating conditions. From the concentrations of fatty acids, total unsaturated fatty acids (UFA), monounsaturated fatty acids (MUFA), polyunsaturated fatty acids (PUFA), saturated fatty acids (SFA), and n-6/n-3 ratios were determined. Hypercholesterolemic fatty acids, hypocholesterolemic fatty acids, atherogenic index (AI) and linoleic acid/alpha-linolenic acid (LA/ALA) ratios were also calculated. The atherogenic index (AI) was calculated as IA-Σ (C12:0, C14:0 and C16:0)/UFA).

#### 2.3.5. Genotyping

The primers presented in Table 1 were used to amplify fragments of the *FADS2* and *FABP4* genes. PCR reactions (20 µL) were performed in an AG22331 thermocycler (Eppendorf, Hamburg, Germany). The mixture consisted of 1x PCR buffer, 2.5 mM MgCl_2_, 0.2 mM dNTP, 0.2 µM of each primer, 1.2 U Taq DNA polymerase I (Thermo Scientific, Vilnius, Lithuania), and 150–200 ng DNA. Thermal program for each gene included the following steps: initial denaturation at 94 °C for 2 min, 35 cycles of proper denaturation at 94 °C, primer annealing (Table 1), elongation at 72 °C for 30 s, and final elongation at 72 °C for 5 min. Fragments of the amplified genes were cut with restriction enzymes for 4 h (Table 1) and separated on a 2% agarose gel (Agarose Type I-A, Sigma Aldrich, St. Louis, MO, USA) in 1x TBE buffer; visualization was performed using BOX Chemi XR5 (Syngene, Cambridge, England).

### 2.4. Statistical Analysis

The frequencies of *FADS2* and *FABP4* genotypes were determined and it was verified with ch2-test whether their distributions conformed to those expected (according to the Hardy–Weinberg law). A statistical evaluation was performed to compare the carcass and meat quality traits between chickens of different *FADS2* and *FABP4* genotypes using the least-square method of the GLM procedure (Statistica v. 11PL, StatSoft Polska, Krakow, Poland). The normality of data distribution was tested using the Shapiro–Wilk test and homogeneity of variance was tested by the Levene’s test. The linear model used in statistical analysis of the data included *FADS2* and *FABP4* genotypes and interactions between experimental factors. No *FADS2* × *FABP4* interactions were found, so only genotype effects are reported. A detailed comparison of the least-square means (LSM) for the analyzed genotypes was done using the Tukey–Kramer test. *p* < 0.05 was considered statistically significant. The date are expressed as LSM ± SE (standard error) of the means.

## 3. Results

### 3.1. Analysis of FADS2 and FABP4 Gene Polymorphisms

PCR reaction amplified a 283 bp fragment of the *FADS2* gene promoter. The identification of the g.4290 C>G polymorphism revealed the presence of 3 possible genotypes. The prevalence of broilers with the CC (0.55) compared to GC genotype (0.38) was demonstrated. The allele with G substitution occurred with a frequency of 0.26 (Table 1). A 477 bp fragment of the *FABP4* gene was also amplified and the identification of the g.285 C>T polymorphism revealed the presence of 3 genotypes as well. The most common were the CT and CC genotypes with a frequency of 0.49 and 0.31, respectively. The T allele with the g.285 C>T substitution occurred with a frequency of 0.45 (Table 1). The studied population was in Hardy–Weinberg equilibrium at both analyzed loci.

### 3.2. Influence of the FADS2 and FABP4 Gene Polymorphisms on Carcass and Meat Quality

#### 3.2.1. *FADS2*

The effect of the polymorphism on final body weight and post-slaughter traits is presented in Table 2. The influence of the polymorphism on abdominal fat content was demonstrated. Less fat (1.13%) was found in birds with the CG vs. CC genotype (*p* < 0.05). When examining the influence of the polymorphisms on chicken meat quality parameters, differences were shown only for the thermal loss index, with a higher value by 7% (*p* < 0.05) in individuals with the heterozygous CG vs. CC genotype.

#### 3.2.2. *FABP4*

No influence of the genotype in the *FABP4 locus* was found for any of the studied features of the carcass. Considering the meat quality characteristics, the meat of birds with the TT vs. CC genotype had 10% higher values only in the case of thermal losses (*p* < 0.05).

### 3.3. Effect of Polymorphism in the FADS2 and FABP4 Genes on the Fatty Acid Profile in Pectoralis Superficialis Muscle

The effect of SNPs on the fatty acid profile of intramuscular fat of male chicken pectoral muscles is presented in Table 3. A statistically significant effect of the genotype at the *FADS2 locus* on saturated (SFA) and unsaturated (UFA) fatty acid contents was found. The presence of SNPs caused a decrease in the content of C14:0 acids and an increase in C18:0. At the same time, the content of C14:1, C16:1, and C18:1 monounsaturated acids decreased, which resulted in differences in total monounsaturated fatty acids (MUFA) between the CC and GG genotypes, as well as the CC and CG genotypes (*p* < 0.01).

In GG vs. CC individuals, an increase in the content of C20:2 acid (*p* < 0.01) was demonstrated. In the intramuscular fat of the GG vs. CC genotype, the ARA acid values were doubled (C20:4n-3, *p* < 0.05) and EPA were higher by 47% (C20:5, *p* < 0.01). A twofold increase in docosapentaenoic fatty acid (DPA) (C 22:5 n-6) content was also found in CG and GG vs. CC. The highest DHA (C22:6) values were recorded for GG vs. CC (*p*< 0.01). A lower content in the meat of the GG vs. CC genotype was noted only for ALA and conjugated linoleic (CLA) acids. The values of the LA/ALA ratio were lower in male chickens with the GG vs. CC genotype (*p* < 0.05). There was also a decrease in the quantity of total UFAs for the GG vs. CC genotype (*p* < 0.01), while no effect of the genotype on the total content of PUFA or hypo- and hypercholesterolemic acids (DFA and OFA) was found.

The analysis of the influence of the genotype at the g.285 C>T *locus* on the fatty acid profile in *pectoralis superficialis* muscle showed differences in the content of individual acids. This pertained to C18:1, C18:2n-6 (LA) and C22:6n-3 (DHA) acids (Table 4). For C18:1 acid, the values were higher in the CT genotype (*p* < 0.05) vs. CC genotype, and for LA, a difference (*p* < 0.05) was determined between CC vs. TT. The presence of the T allele lowered DHA content, and the CC genotype showed higher values than the TT genotype (*p* < 0.05).

The analysis of the content of UFA as well as MUFA and PUFA showed no differences, similarly for the total of OFA and DFA.

## 4. Discussion

Variation analysis in the promoter of the *FADS2* gene, *locus* g.4290 C>G in Ross 308 broiler chickens demonstrated the highest frequency of the CC genotype, which resulted in a high frequency of the wild-type C allele (0.74). It should be emphasized that the results of genotype distribution in commercial line Ross 308 were similar to those published in the Chinese Gushi and Anak breeds, where CC occurred with a high frequency of 0.63. The studied polymorphism did not affect the final weight of the birds, which did not confirm the results demonstrated in Chinese breeds by Zhu et al. [5].

It should be noted that the occurrence of the g.4290 C>G substitution led to statistically significant changes in the fatty acid profile of the *pectoralis superficialis* muscle. The *FADS2* polymorphism differentiated SFA, MUFA, and UFA contents. The presence of SNPs caused an increase in SFA in G (CG) allele carriers, which was mainly due to the increase in the content of saturated stearic acid (C18:0). The decrease in MUFA content in GC and GG birds was mainly the result of lower oleic acid content (C18:1n-9). Changes in the content of individual n-3 and n-6 polyunsaturated acids resulted in the improvement of meat functional parameters. It should be emphasized that the content of EPA (C20:5n-3), ARA (C20:4n-6), DPA (C22:5n-6), and docosatetraenoic acid (C22:4n-6) was double in *pectoralis superficialis* muscle in GG male chickens compared to CC male chickens. As regards EPA, an acid that determines the proper functioning of the human nervous system and vision, as well as docosahexaenoic acid, the presence of the g.4290 C>G SNP in the *FADS2* gene led to favorable changes in the content of these acids in broiler *pectoralis superficialis* muscle. The results of Chen et al. [21] confirmed the observed effect of g.4290 C>G on a decrease in oleic acid, accompanied by an increase in EPA, DPA, and DHA.

No statistically significant differences were shown for LSM, the sum of DFA and OFA fatty acids in *pectoralis superficialis* muscle, but it was observed that the GG birds had a preferably lower DFA content. The tested polymorphism also influenced the IA index value and caused its statistically significant increase in birds with the GG genotype.

The results confirmed the findings of Zhu et al. [5], who identified the occurrence of SNP g.4290 C>G and g.4291 C>A in the *FADS2* gene in F2 Gushi-Anak chickens. They demonstrated a significant relationship of both SNPs with the fatty acid profile in birds’ muscles. The increase in C18:3 and C20:4 contents described by them in the meat of chickens with the GG genotype was consistent with the results obtained in the present study. The influence of the genotype of the *FADS2* gene on ARA and DHA contents in *pectoralis superficialis* muscle, demonstrated in the current study, was not related to the differences in the final weight of chickens with different genotypes. This result was not confirmed by the results of Boschetti et al. [1], who observed different predispositions to synthesis and accumulation of n-3 long-chain LC-PUFA in the pectoral muscles of birds with different growth rates (slow, medium, and fast growing chickens). The relative liver expression of the genes encoding delta-6 and delta-5 fatty acid desaturase proteins, as well as the activity of enzymes in the pectoral muscle, was shown to be dependent on chicken growth rate [1]. Similar results were presented by Sirri et al. [22], who showed that ARA and DHA contents in the pectoral muscles of slow-growing male chickens was higher than in fast- and medium-growing male chickens. It was also demonstrated that the muscle acid profile could be modified, for example, by castration. Male chickens, compared to capons, had a higher ARA, DHA, and DPA contents in the pectoral and leg muscles [23].

The presence of the tested SNP in the *FADS2* gene improved the fatty acid profile of intramuscular fat. These include ARA, DHA, and EPA, which are very important in human nutrition. DHA is essential for the proper functioning of organs and cells. It is present in the highest concentrations in phospholipids of the retina and brain, as well as in the vicinity of nerve synapses [24]. EPA is a substrate in DHA synthesis, and together with ARA, it determines the synthesis of eicosanoids, important in the regulation of such processes as immunity, inflammation, reproduction, and development [4,25]. It was shown that the *FADS2* polymorphism changed ARA and DHA contents in the meat of one of three most popular chicken breeds in Japan—Hinai-Jidori. ARA content may also be important for meat sensory characteristics, as meat of birds with a higher ARA content is tastier [26].

The present research is all the more important as it was carried out on one of the most popular broiler lines—Ross 308. This line is the result of an ongoing genetic selection program. It is characterized by an excellent rate of weight gain, high disease resistance, optimal feed consumption, good survival, and carcass quality. In this study, no influence was found of g.4290 C>G on the n-6/n-3 PUFA ratio, which is essential for the health quality of meat and usually exceeds a value of 4, considered optimal by nutritionists [27]. On the other hand, lower values of the LA/ALA ratio were recorded in the pectoral muscles of birds with the GG genotype, which should be considered a favorable phenomenon in terms of human nutrition [28].

Studies conducted on large farm animals suggested the influence of the *FADS2* and *FABP4* gene polymorphisms on the quality of cattle meat. Comparison of the sequences of the conserved promoter regions in the *FADS2* and *FABP4* genes between Japanese Black and Holstein individuals confirmed the presence of SNPs determining the characteristics of fat tissue, including the assessment of meat marbling. The authors suggest that these SNPs can be used as genetic markers to improve beef quality. Matsumoto et al. [15] also demonstrated sequence variation in the *FADS2* gene in Canadian Holstein cattle and confirmed the relationship between polymorphisms and fatty acid (dihomo-ɣ-linolenic acid, arachidonic and eicosapentaenoic acid) contents in cow milk. SNPs in the studied regions significantly affected the expression of genes or the function of the resulting protein products and determined the level of important production traits in farm animals [14].

In the studied bird population, three genotypes were found at the g.248 C>T *locus* of the *FABP4* gene, with the CT genotype (0.49) being the most frequent and TT the least frequent (0.19). The wild-type C allele occurred with the highest frequency (0.55). A similar distribution of genotypes was demonstrated in Ross broilers in the study of Maharani et al. (2011) [29], but it should be noted that genotype distribution could depend on breed or lineage, as the TT genotype was most frequent in Korean chickens [30].

The analysis of the effect of the g.285 C>T polymorphism on weight gain and carcass value showed no statistically significant differences between genotypes, confirming the results obtained by Cahyadi et al. [30]. As in the abovementioned publication, it was also observed in the present study that the lowest values of weight gain were observed in birds with the CC genotype, but these differences were not statistically significant. There was no effect of the *FABP4* gene polymorphism on meat quality either, and only an increase by 10% (*p* < 0.05) in the thermal loss index in CC vs. TT chickens was observed.

The results of this study, showing no effect of SNP in *FABP4* on abdominal fat content in chickens, differ from the results of Wang et al. [31] in the broiler and Baier lines. The authors identified the p.Ser89Asn polymorphism in exon 3 of the *FABP4* gene and found that abdominal fat content at 12 weeks of age was lower in the Baier race (0.89%) than in the broiler line (3.74%). The effect of C51T substitution in exon 1 of the *FABP4* gene on the content of intramuscular fat in the pectoral muscles of the Hetian black and three-yellow chicken breeds was demonstrated [32,33].

The important role of the *FABP4* protein in the transport and metabolism of fatty acids should be emphasized. The *FABP4* protein, by binding with oleic acid, interacts with the hormone-sensitive lipase (HSL), responsible for lipolysis in tissues [34]. It has been found that *FABP4* secreted from macrophages is responsible for cholesterol transport, atherosclerotic lesions, and anti-inflammatory activity; these processes are associated with the PPARα factor [35]. Mice lacking the *FABP4* (*FABP4^−/−^*) and *E-FABP* (*E-FABP^−/−^*) genes had a higher content of fatty acids in plasma compared to wild-type animals. Changes in the fatty acid profile were associated with alterations in the expression of these genes and reduced the activity of stearoyl-coenzyme A (SCD) desaturase, involved in the synthesis of MUFA, phospholipids, triglycerides, and cholesterol esters [36,37].

The GLM model used to analyze the effect of the *FABP4* polymorphism on FA composition in *pectoralis superficialis* muscle showed the influence of g.285 C>T on the content of C18:1n-9, C18:2n-6 and DHA. Oleic acid content reached the highest values in individuals with the CT genotype, and linoleic acid content in the TT genotype. The occurrence of the T allele lowered the DHA value, which is essential in nutritional products, especially recommended for children, as it is associated with improvement in brain growth and function [38]. A similar result of DHA decrease in the meat of TT genotype broilers was reported by Maharani et al. [29]. In the present study, no effect of the g.285 C>T substitution on the content of arachidonic acid C20:4 was found, as demonstrated by other authors [29]. There was a tendency of higher MUFA, PUFA and hypocholesterolemic DFA acid contents in birds with the g.285 C>T substitution, but these differences were not statistically significant.

## 5. Conclusions

The g.4290 C>G polymorphism was identified in the *FADS2* gene *locus* in Ross 308 male chickens. The g.4290 C>G substitution occurred in the study population with a 0.26 frequency and the most common genotypes were CG heterozygotes (38%) and CC homozygotes (55%). The studied polymorphism exerted an effect on the *pectoralis superficialis* muscle fatty acid profile; it caused an increase in SFA and a decrease in MUFA, as well as an increase in the content of DHA and EPA acids, important in human nutrition. The investigated population of commercial broiler chickens at loci *FADS2* and *FABP4* was in H-W equilibrium. For *FADS2*, a statistically significant effect of SNP on the fatty acid profile of the *pectoralis superficialis* muscle was found. Both studied genes play a key role in the metabolism of fatty acids in animal adipose tissue, so there are probably some mechanisms preventing the disturbance of H-W balance, i.e., reducing the importance of selection pressure (if any).

Our results suggest that the *FADS2*/*Rsa*I genotype might be utilized in the selection of valuable chicken quality meat traits, particularly on the *pectoralis superficialis* muscle fatty acid profile.

## Figures and Tables

**Table 1:** Details of single-nucleotide polymorphism markers, genes, primers.

Gene	Chr	SNP	Primers (5′ to 3′)	Ta (°C)	Enzyme	Tt (°C)	Product PCR/Genotype
*FADS2*	5	g.4290 C>G	F: TGAACTGAAGCGTTTGATGG R: TGGCTTTCTTGGCAATAGG	57.5	*Rsa*I	37	283 bp CG (283, 156, 127 bp) CC(156 i 127 bp) GG (283 bp)
*FABP4*	2	g.285 C>T	F: TGTGACTACTGGCAAAGGA R: TTCCTCCCAGTCAAGCTTTC	63.5	*Taq*I	65	477 bp TT (477 bp) CT (477, 242, 235 bp) CC (242, 235 bp)

Chr, Chromosome; *FADS2*, fatty acid desaturase 2 (NCBI Ref. Seq. NW_001471700); *FABP4*, fatty acid binding protein 4 (NCBI Ref. Seq. NC_006089); PCR, polymerase chain reaction; Ta, annealing temperature; Tt, pickling temperature; SNP, single-nucleotide polymorphism.

**Table 2:** Least squares ± S.E. for broiler rearing performance, dressing percentage, carcass quality and *pectoralis superficialis* muscle quality parameters of *FADS2* and *FABP4* polymorphism.

Traits	*FADS2* Genotype			*FABP4* Genotype		
	CC (0.55) *	CG (0.38)	GG (0.07)	CC (0.31)	CT (0.49)	TT (0.20)
*Rearing performance, dressing percentage, and carcass quality*						
BW (kg)	3.44 ± 0.28	3.52 ± 0.27	3.56 ± 0.40	3.48 ± 0.34	3.50 ± 0.27	3.49 ± 0.43
DRP (%)	77.99 ± 0.22	78.08 ± 0.26	78.59 ± 0.26	78.01 ± 0.23	77.83 ± 0.23	78.85 ± 0.40
Cr (kg)	2.58 ± 0.22 ^A^	2.62 ± 0.21	2.69 ± 0.28 ^B^	2.61 ± 0.34	2.62 ± 0.26	2.65 ± 0.42
BRM (%)	31.86 ± 0.24	32.23 ± 0.21	31.51 ± 0.77	32.02 ± 0.31	31.89 ± 0.25	32.10 ± 0.49
LGM (%)	18.36 ± 0.11	19.23 ± 0.15	19.0 ± 0.14	19.88 ± 0.05	19.56 ± 0.05	19.75 ± 0.05
LGB (%)	4.07 ± 0.26	4.37 ± 0.13	4.15 ± 0.30	4.55 ± 0.08	4.58 ± 0.07	4.45 ± 0.09
G (%)	3.83 ± 0.05	3.78 ± 0.06	3.71 ± 0.08	3.76 ± 0.06	3.87 ± 0.06	3.73 ± 0.08
AF (%)	1.34 ± 0.05 ^b^	1.13 ± 0.04 ^a^	1.28 ± 0.11	1.16 ± 0.06	1.37 ± 0.06	1.19 ± 0.08
*Pectoralis superficialis muscle quality parameters*						
pH 24 h	5.80 ± 0.01	5.81 ± 0.01	5.77 ± 0.02	5.78 ± 0.02	5.80 ± 0.01	5.84 ± 0.02
Colour L*	58.45 ± 0.39	59.32 ± 0.37	59.05 ± 0.47	58.76 ± 0.49	59.13 ± 0.34	58.72 ± 0.46
a*	10.48 ± 0.23	10.15 ± 0.21	10.66 ± 0.36	10.38 ± 0.33	10.36 ± 0.20	10.37 ± 0.31
b*	9.28 ± 0.18	9.56 ± 0.20	9.73 ± 0.32	9.64 ± 0.28	9.31 ± 0.16	9.75 ± 0.22
TL (%)	24.74 ± 0.47 ^b^	26.51 ± 0.46 ^a^	25.32 ± 0.68	24.69 ± 0.59 ^a^	25.14 ± 0.40 ^ab^	27.46 ± 0.69 ^b^
WBS (N)	18.33 ± 0.39	19.13 ± 0.63	18.55 ± 0.83	18.58 ± 0.66	18.04 ± 0.40	19.60 ± 0.69

* Genotype frequency; ^A–B^ Means with different letters within rows differ significantly (*p* < 0.01); ^a–b^ Means with different letters within rows differ significantly (*p* < 0.05); BW, body weight; DRP, dressing percentage without giblets; Cr, carcass weight; G, proportion of giblets in the carcass; BRM, proportion of breast muscles in the carcass; LGM, proportion of leg muscles in the carcass; LGB, proportion of leg bones in the carcass; AF, proportion of abdominal fat in the carcass; L*, a*, b*, muscle colour: L* (lightness), a* (redness) and b* (yellowness); TL, thermal loss; WBS, Warner–Bratzler shear force.

**Table 3:** Association between g.285 C>T SNP of *FABP4* gene and g.4290 C>G SNP of *FADS2* with FA profile of *pectoralis superficialis* muscle (TA%).

Traits	*FADS2* Genotype			*FABP4* Genotype		
	CC	CG	GG	CC	CT	TT
14:0	1.36 ± 0.01 ^A^	1.25 ± 0.02 ^B^	1.29 ± 0.02	1.32 ± 0.02	1.4 ± 0.2	1.40 ± 0.03
14:1	0.29 ± 0.01 ^A^	0.24 ± 0.01 ^B^	0.24 ± 0.01 ^B^	0.27 ± 0.01	0.29 ± 0.01	0.28 ± 0,02
15:0	0.21 ± 0.01	0.213 ± 0.00	0.22 ± 0.01	0.21 ± 0.01	0.23 ± 0.02	0.21 ± 0.01
16:0	24.39 ± 0.14	25.83 ± 0.69	26.18 ± 0.64	25.6 ± 0.5	24.0 ± 0.5	23.6 ± 0.3
16:1	5.14 ± 0.09 ^A^	4.42 ± 0.13 ^B^	4.17 ± 0.10 ^B^	4.70 ± 0.15	4.98 ± 0.12	5.06 ± 0.12
17:0	0.31 ± 0.01	0.34 ± 0.01	0.34 ± 0.01	0.32 ± 0.01	0.30 ± 0.01	0.31 ± 0.01
17:1	0.31 ± 0.01 ^A^	0.28 ± 0.01 ^B^	0.27 ± 0.01 ^B^	0.28 ± 0.01	0.29 ± 0.01	0.29 ± 0.01
18:0	8.36 ± 0.18 ^A^	9.59 ± 0.22 ^B^	10.16 ± 0.20 ^B^	9.43 ± 0.18	8.79 ± 0.20	8.81 ± 0.29
18:1	41.91 ± 0.37 ^A^	38.78 ± 0.62 ^B^	37.68 ± 0.43 ^B^	39.75 ± 0.34	40.9 ± 0.37	40.32 ± 0.47
18:2	13.89 ± 0.19	13.94 ± 0.22	13.72 ± 0.21	13.75 ± 0.16 ^a^	13.77 ± 0.20 ^b^	14.15 ± 0.17 ^ab^
18:3n-3	1.15 ± 0.06 ^a^	1.01 ± 0.04	0.95 ± 0.04 ^b^	1.15 ± 0.01	1.1 ± 0.03	1.23 ± 0.02
CLA	0.21 ± 0.00 ^A^	0.21 ± 0.00 ^A^	0.18 ± 0.00 ^B^	0.19 ± 0.00 ^a^	0.20 ± 0.00 ^ab^	0.21 ± 0.00 ^b^
20:0	0.05 ± 0.00 ^a^	0.06 ± 0.01	0.06 ± 0.04 ^b^	0.06 ± 0.00	0.06 ± 0.00	0.05 ± 0.00
20:1	0.20 ± 0.00	0.21 ± 0.01	0.21 ± 0.01	0.20 ± 0,00	0.21 ± 0.00	0.20 ± 0.00
20:2	0.11 ± 0.01 ^Aa^	0.15 ± 0.01 ^b^	0.18 ± 0.01 ^B^	0.14 ± 0.01	0.13 ± 0.00	0.13 ± 0.00
20:3n-6	0.19 ± 0.01 ^Aa^	0.28 ± 0.03 ^b^	0.35 ± 0.02 ^B^	0.26 ± 0.01	0.23 ± 0.01	0.24 ± 0.01
20:4	0.98 ± 0.07 ^Aa^	1.69 ± 0.23 ^b^	2.02 ± 0.17 ^B^	1.43 ± 0.03	1.24 ± 0.02	1.34 ± 0.01
20:5n-3	0.06 ± 0.01 ^Aa^	0.10 ± 0.01 ^b^	0.12 ± 0.01 ^B^	0.08 ± 0.00	0.08 ± 0.00	0.07 ± 0.00
22:4n-6	0.22 ± 0.01 ^Aa^	0.40 ± 0.05 ^b^	0.50 ± 0.04 ^B^	0.35 ± 0.03	0.28 ± 0.02	0.33 ± 0.03
22:5	0.25 ± 0.01 ^Aa^	0.41 ± 0.05 ^b^	0.47 ± 0.04 ^B^	0.36 ± 0.04	0.33 ± 0.03	0.32 ± 0.4
22:6n-3	0.19 ± 0.01 ^Aa^	0.29 ± 0.03 ^b^	0.36 ± 0.03 ^B^	0.30 ± 0.02	0.26 ± 0.02	0.26 ± 0.02

Values in table represent least squares mean (LSM ± SE); CLA, conjugated linoleic acid. ^A–B^ Means with different letters within rows differ significantly (*p* < 0.01); ^a–b^ Means with different letters within rows differ significantly (*p* < 0.05).

**Table 4:** Least-squares ± S.E. of health quality parameters based on the FA profile (TA%).

Traits	*FADS2* Genotype			*FABP4* Genotype		
	CC	CG	GG	CC	CT	TT
SFA	34.75 ± 0.31 ^Aa^	37.35 ± 0.89 ^b^	38.34 ± 0.82 ^B^	36.58 ± 0.47	35.44 ± 0.56	35.42 ± 0.86
UFA	65.14 ± 0.31 ^Aa^	62.50 ± 0.88 ^b^	61.49 ± 0.81 ^B^	63.28 ± 0.46	64.43 ± 0.66	64.46 ± 0.85
MUFA	47.87 ± 0.40 ^A^	43.97 ± 0.62 ^B^	42.60 ± 0.42 ^B^	45.03 ± 0.407	46.53 ± 0.46	45.96 ± 0.62
PUFA	17.27 ± 0.28	18.53 ± 0.54	18.89 ± 0.53	18.04 ± 0.37	17.69 ± 0.37	18.28 ± 0.33
n-6/n-3	9.76 ± 0.33	9.53 ± 0.35	9.51 ± 0.31	9.82 ± 1.26	9.95 ± 1.44	9.9 ± 1.33
DFA	73.51 ± 0.14	72.09 ± 0.69	71.65 ± 0.64	72.72 ± 0.36	73.23 ± 0.39	73.27 ± 0.60
OFA	26.39 ± 0.14	27.76 ± 0.71	28.17 ± 0.65	27.14 ± 0.36	26.65 ± 0.39	26.61 ± 0.60
Index IA	0.39 ± 0.00 ^a^	0.44 ± 0.01	0.45 ± 0.01 ^b^	0.42 ± 0.01	0.41 ± 0.01	0.41 ± 0.01
LA/ALA	14.84 ± 0.52 ^a^	14.25 ± 0.62	12.88 ± 0.57 ^b^	14.35 ± 2.42	13.89 ± 2.42	13.47 ± 2.53

SFA = ∑ (C10:0, C12:0, C14:0, C15:0, C16:0, C17:0, C18:0, C20:0); UFA = MUFA + PUFA, MUFA = ∑ (C14:1, C16:1, C17:1, C18:1, C20); PUFA–∑ (C18:2, CLA, C18:3, C20:2, C20:3, C20:4, C20:5, C22:4, C22:5 and C22:6). DFA (hypocholesterolemic fatty acids) = UFA + C18:0; OFA (hypercholesterolemic fatty acids) = SFA–C18:0; PUFA n-6–Σ C20:2, C20:4 and C22:4; PUFA n-3–Σ (C18:3, C20:5, C22:5 and C22:6); PUFA n-3–C18:3, IA (index of atherogenicity) = (Σ C12:0, C14:0 and C16:0)/UFA, LA/ALA = linoleic acid/alpha-linolenic acid. ^A–B^ Means with different letters within rows differ significantly (*p* < 0.01); ^a–b^ Means with different letters within rows differ significantly (*p* < 0.05).

## Data Availability

Data are available upon request from the corresponding author.

## References

[1] Boschetti E., Bordoni A., Meluzzi A., Castellini C., Dal Bosco A., Sirri F. (2016). Fatty acid composition of chicken breast meat is dependent on genotype-related variation of FADS1 and FADS2 gene expression and desaturating activity. Animal.

[2] Jing M., Gakhar N., Gibson R.A., House J.D. (2013). Dietary and ontogenic regulation of fatty acid desaturase and elongase expression in broiler chicken. Prostsglandins Leukot. Essent. Fat Acids.

[3] Kaczor U., Borys B., Pustkowiak H. (2010). Effect of intensive fattening of lambs with forages on the fatty acid profile of intramuscular and subcutaneous fat. Czech J. Anim. Sci..

[4] Rikimaru K., Egawa Y., Yamaguchi S., Takahashi H. (2016). Association of chicken fatty acid desaturase 1 and 2 gene single-nucleotide polymorphisms with the fatty acid composition of thigh meat in Japanese Hinai-dori crossbred chickens. J. Fish. Livest. Prod..

[5] Zhu S.K., Tian Y.D., Zhang S., Chen Q.X., Wang Q.Y., Han R.L., Kang X.T. (2014). Adjacent SNPs in the transcriptional regulatory region of the FADS2 gene associated with fatty acid and growth traits in chickens. Genet. Mol. Res..

[6] Burdge G. (2019). Is essential fatty acid inconversion an important source of PUFA in humans?. Br. J. Nutr..

[7] Cho H.P., Nakamura M.T., Clark S.D. (1999). Cloning, expression, and nutritional regulation of the mammalian Delta -6 desaturase. J. Biol. Chem..

[8] Harris W.S., Lemke S.L., Hansen S.N., Goldstein D.A., DiRienzo M.A., Su H., Nemeth M.A., Taylor M.L., Ahmed G., George C. (2008). Stearidonic acid-enriched soybean oil increased the omega-3 index, an emerging cardiovascular risk marker. Lipids.

[9] Simopoulos A.P. (2011). Importance of the omega-6/omega-3 balance in health and disease: Evolutionary aspects of diet. World Rev. Nutr. Diet..

[10] Zimmerman A.W., Veerkamp J.H. (2002). New insights into the structure and function of fatty acid-binding proteins. Cell Mol. Life Sci..

[11] Kucharski M., Kaczor U. (2017). Fatty acid binding protein 4 (FABP4) and the body lipid balance. Folia Biol..

[12] Hertzel A.V., Bernlohr D.A. (2010). The mammalian fatty acid-binding protein multigene family: Molecular and genetic insights into function. Trends Endocrinol. Metab..

[13] Furuhashi M., Saitoh S., Shimamoto K., Miura T. (2014). Fatty Acid-Binding Protein 4 (FABP4): Pathophysiological Insights and Potent Clinical Biomarker of Metabolic and Cardiovascular Diseases. Clin. Med. Insights Cardiol..

[14] Ibeagha-Awemu E.M., Akwanji K.A., Beaudoin F., Zhao X. (2014). Associations between variants of FADS genes and omega-3 and omega-6 milk fatty acids of Canadian Holstein cows. BMC Genet..

[15] Matsumoto H., Nogi T., Tabuchi I., Oyama K., Mannen H., Sasazaki S. (2014). The SNPs in the promoter regions of the bovine FADS2 and FABP4 genes are associated with beef quality traits. Livest. Sci..

[16] Blecha I.M.Z., Siqueira F., Ferreira A.B.R., Feijó G.L.D., Torres Junior R.A.A., Medeiros S.R., Sousa I.I., Santiago G.G., Ferraz A.L.J. (2015). Identification and evaluation of polymorphisms in FABP3 and FABP4 in beef cattle. Genet. Mol. Res..

[17] Gao Y., Zhang Y.H., Zhang S., Li F., Wang S., Dai L., Zhang J.B. (2011). Association of A-FABP gene polymorphism in intron 1 with meat quality traits in Junmu No. 1 white swine. Gene.

[18] Goszczynski D.E., Papaleo-Mazzucco J., Ripolia M.V., Villarreal E.L., Rogberg-Muñoz A., Mezzadra C.A., Melucci L.M., Giovambattista G. (2017). Genetic Variation in FABP4 and Evaluation of Its Effects on Beef Cattle Fat Content. Anim. Biotech..

[19] Połtowicz K., Nowak J., Wojtysiak D. (2015). Effect of feed restriction on performance, carcass composition and physicochemical properties of the m. pectoralis superficialis of broiler chickens. Ann. Anim. Sci..

[20] Folch J., Lees M., Stanley G.H.S. (1957). A simple method for the isolation and purification of total lipides from animal tissues. J. Biol. Chem..

[21] Chen X., Wu Y., Zhang Z., Zheng X., Wang Y., Yu M., Liu G. (2019). Effects of the rs3834458 Single Nucleotide Polymorphism in FADS2 on Levels of n-3 Long-chain Polyunsaturated Fatty Acids: A Meta-analysis. Prostaglandins Leukot. Essent. Fat Acids..

[22] Sirri F., Castellini C., Bianchi M., Petracci M., Meluzzi A., Franchini A. (2011). Effect of Fast-, Medium- and Slow-Growing Strains on Meat Quality of Chickens Reared under the Organic Farming Method. Animal.

[23] Sirri F., Bianchi M., Petracci M., Meluzzi A. (2009). Influence of Partial and Complete Caponization on Chicken Meat Quality. Poult. Sci..

[24] Lagarde M., Guichardant M., Picq M., Michaud S., Doutheau A. (2008). Method for Preparing Acetyl, Docosahexaenoyl-Glycerophosphocholine and Use Thereof for the Delivery of Polyunsaturated Fatty Acids. European Patent.

[25] Cha Y.I., Solnica-Krezel R.N., DuBois R.N. (2006). Fishing for prostanoids: Deciphering the developmental functions of cyclooxygenase-derived prostaglandins. Dev. Biol..

[26] Takahashi H., Rikimaru K., Kiyohara R., Yamaguchi S. (2012). Effect of Arachidonic Acid-Enriched Oil Diet Supplementation on the Taste of Broiler Meat. Asian Australas J. Anim. Sci..

[27] Bartoň L., Marounek M., Kudrna V., Bureš D., Zahradová R. (2007). Growth performance and fatty acid profiles of intramuscular and subcutaneous fat from Limousin and Charolais heifers fed extruded linseed. Meat Sci..

[28] Wijendran V., Hayes K.C. (2004). Dietary n-6 and n-3 fatty acid balance and cardiovascular health. Annu. Rev. Nutr..

[29] Maharani D., Park H.B., Jung Y., Jung S., Jo C., Lee J.H. (2011). Investigation of SNPs in FABP3 and FABP4 genes and their possible relationships with fatty acid composition in broiler. Korean J. Poult. Sci..

[30] Cahyadi M., Seo D., Choi N., Jin S., Maharani D., Heo K.N., Kang B.S., Jo C., Lee J.H. (2013). FABP3 and FABP4 Genes Are the Potential Candidates for Body Weights in Korean Native Chicken. Korean J. Poult. Sci..

[31] Wang Q., Guan T., Li H., Bernlohr D.A. (2009). A Novel Polymorphism in the Chicken Adipocyte Fatty Acid- Binding Protein Gene (FABP4) That Alters Ligand-Binding and Correlates with Fatness. Comp. Biochem. Physiol. B Biochem. Mol. Biol..

[32] Wang Q., Li H., Li N., Leng L., Wang Y., Tang Z. (2006). Identification of single nucleotide polymorphism of adipocyte fatty acid-binding protein gene and its association with fatness traits in the chicken. Poult. Sci..

[33] Wang Y., Chen H., Han D., Chen Y., Muhatai G., Kurban T.J. (2016). Correlation of the A-FABP Gene Polymorphism and mRNA Expression with Intramuscular Fat Content in Three-Yellow Chicken and Hetian-Black Chicken. Anim. Biotech..

[34] Storch J., Thumser A.E. (2008). Tissue-specific functions in the fatty acid-binding protein family. J. Biol. Chem..

[35] Makowski L., Brittingham K.C., Reynolds J.M., Suttles J., Hotamisligil G.S. (2005). The fatty acid binding protein, aP2, coordinates macrophage cholesterol trafficking and inflammatory activity. J. Biol. Chem..

[36] Furuhashi M., Hotamisligil G.S. (2008). Fatty acid-binding proteins: Role in metabolic diseases and potential as drug targets. Nat. Rev. Drug Discov..

[37] Ntambi J.M. (1995). The regulation of stearoyl CoA desaturase (SCD). Prog. Lipid Res..

[38] Horrocks L.A., Yeo Y.K. (1999). Health benefits of docosahexaenoic acid (DHA). Pharmacol. Res..

